# Acute injuries in male elite ice hockey players. A prospective cohort study

**DOI:** 10.1016/j.jsampl.2024.100068

**Published:** 2024-07-05

**Authors:** Jussi Hirvelä, Markku Tuominen, Olavi Airaksinen, Timo Hänninen, Niklas Lindblad, Hilkka Ryhänen, Jukka Tikanto, Jari Parkkari

**Affiliations:** a Tampere University, The Faculty of Medicine and Health Technology, Tampere, Finland; b Medisport Ltd, Tampere, Finland; c University of Eastern Finland, Kuopio, Finland; d Tampere Research Center of Sports Medicine, UKK Institute, Tampere, Finland; e Terveystalo Turku Sport Clinic, Turku, Finland; f Satasairaala, Pori, Finland; g Department of Otorhinolaryngology, Oulu University Hospital, Oulu, Finland; h Faculty of Sport and Health Sciences, University of Jyväskylä, Jyväskylä, Finland; i University of Helsinki, Finland

**Keywords:** Ice hockey, Injury, Epidemiology, Concussion

## Abstract

**Background:**

In Finland, elite level ice hockey injury studies have not been conducted since 1990s. Epidemiological data is needed for developing injury prevention. The aim of this study was to calculate the injury incidences and to describe details of the injuries in the men's elite-level ice hockey league in Finland (Liiga).

**Methods:**

During the three seasons of 2017–2020, injuries from eight Liiga teams were documented by team physicians to a digitalized injury-reporting system. All acute injuries requiring medical attention or causing a player's time loss were reported. Injury incidences were calculated, and injury details were described.

**Results:**

Overall, 326 injuries occurred in 1147 matches, comprising an injury rate (IR) of 12.9 per 1000 player-games. The head, including face, was the most commonly injured body part (IR 4.7 per 1000 player-games; 36.2% of the injuries), followed by the upper extremity (IR 3.5; 27.3%) and lower extremity (IR 3.3; 25.8%). Concussions were more frequent (IR 1.9) than knee (IR 1.6) or shoulder (IR 1.2) injuries. Body checking (31.5%) was the most common mechanism of injury, and contusion (29.3%) was the most typical diagnosis. The player's time loss was over three weeks in 17.7% of all injuries, of which mostly were knee (23.1%), hand (21.2%) and shoulder (13.5%) injuries.

**Conclusions:**

The injury rate was slightly lower than in other elite-level ice hockey studies and 28.2% lower than in previous studies conducted in Finland in the 1990s. Head injuries were the most common. Concussions persisted as frequent injuries in elite ice hockey.

## Graphical abstract

**Figure:**
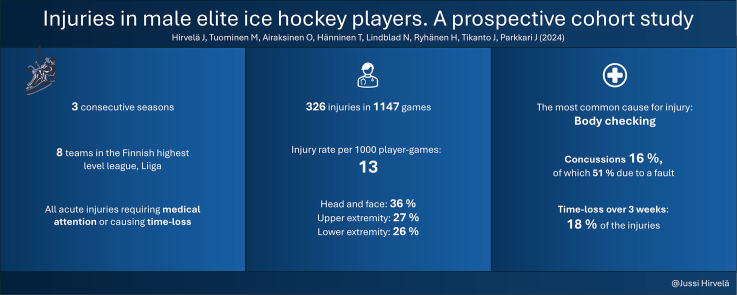

## 1 Introduction

Ice hockey is a high-speed contact sport played especially in Europe and North America. Compared to other winter sports, ice hockey has an average risk of injury [[1], [2], [3]]. Although players wear a comprehensive amount of protective equipment, injuries occur during the intensive 60 ​min game. For example, when colliding at a fast pace with the other players, the puck, or the boards of the ice hockey rink, the players' tissues are prone to acute and repetitive transfers of kinetic energy, which may lead to injuries [4]. In men's ice hockey, the body check (to obstruct another player by tackling him at the torso) is allowed, thereby leading to repetitive collisions between athletes. Rule infringements, such as checking to the head, checking from behind, and high-sticking, also occur and can present injury risks for the players [5]. A consensus statement issued by the International Olympic Committee (IOC) defined an injury as an event either requiring medical attention or causing a player's time loss from the sport [4]. However, an injury can also be defined as any complaint that negatively affects a player's performance.

Previous research in the 2010s examined the incidence of ice hockey injuries in several elite-level leagues and tournaments around the world. For example, McKay et al. [6] documented an injury incidence of 15.6 per 1000 athlete exposures (AE) among NHL players, whereas Tuominen et al. [7] found an injury rate of 14.2 per 1000 player-games in the IIHF World Championships. On the other hand, Brunner et al. [8] recently found time-loss injuries to be as common as 88.6 per 1000 player-game hours in the highest-level ice hockey league in Switzerland, the Swiss National League (SNL). In all three studies, body checking was documented as the most common mechanism of injury [[6], [7], [8]]. McKay's [6] and Brunner's [8] groups have documented hip/groin/thigh region and head being injured most commonly, whereas Tuominen et al. [7] have reported head and face as the most commonly injured body part. Concussions have also been documented as common injuries, accounting for 9.9% of injuries in the IIHF tournaments [7] and 18% in the SNL [8].

However, the most recent epidemiological studies considering Finland's elite-level ice hockey injuries are from the 1990s [9,10]. Over the last two decades, ice hockey has undergone significant changes and improvements in terms of the speed of the game and the quality of the equipment. The safety of the rinks has also improved thanks to the flexible boards that are compulsory in elite-level arenas in Finland. Today, the men's highest-level ice hockey league in Finland, Liiga, consists of 15 teams, each playing 60 games per regular season. The number of players in the squad is 22, and the size of the ice rink surface is comparable to the IIHF specifications [11]. Objective data on modern-day ice hockey injuries are required to improve player safety.

The aim of the present research was to measure the injury incidences and describe the details of the injuries during the three seasons from 2017 to 2020 in Liiga. The current injury rates were also compared with those of previous studies.

## 2 Materials and methods

The research was conducted as a prospective cohort study. Ice hockey injuries were registered during the three seasons 2017–18, 2018–19, and 2019–20 in Liiga, the highest-level ice hockey league in Finland. Only injuries occurring during official matches in the regular season and playoffs were included. Each injury was documented as an Injury Report System (IRS) form in an electrical database by the teams' healthcare staff. The IRS forms recorded a variety of injury details, such as diagnosis, type, mechanism, severity, and possible penalization. Injuries were diagnosed by the team physicians, while team athletic trainers and physiotherapists assisted in documenting the injury reports. The teams were able to update the data afterward. Before every season, a seminar was held, where the teams’ healthcare staff were instructed to use the IRS, and personal accounts were given to the system. The teams were only able to access their own data. All data were registered with the permission of Liiga. All analyses were conducted anonymously.

An ice hockey injury was defined by a strict classification comparable to previous ice hockey studies that considered IIHF tournaments [7]. A combination of acute injuries that required medical attention or caused time loss was registered. Criteria for a time-loss injury were met if the player was unable to continue the game or missed the following game or practice due to the injury. Treatment involving lacerations and all dental injuries, concussions, and fractures were registered. The inclusion criteria were also presented in the web-based monitoring system, where the information was collected. Injuries not meeting the inclusion criteria, e.g., overuse injuries (n ​= ​3) and illnesses (n ​= ​2), were excluded.

The injury incidence was presented per 1000 player-games and per 1000 player-game hours to facilitate comparisons with previous studies on ice hockey and other sports [4]. Each player on the team was at risk of getting injured during the whole game, whether on the ice or not. Even on the substitutes' bench, players can get injured preparing for their next shift. Therefore, our estimates of athlete exposure included all 22 team players in the denominator. The injury rate (IR) per 1000 player-game hours was calculated assuming that both teams had five players and a goalie playing on the rink for the whole 60 ​min of the game. Possible overtime and penalties were not considered in the total exposure time. Athlete exposure (AE) was estimated based only on injuries sustained during official matches in the regular season and playoffs. The number of games was calculated by aggregating all the games played by every individual team participating in the research. Hence, the number of teams used in the formula's divisor was one [7].

$$\text{IR}\;\text{per}\;1000\;\text{player}-\text{game}\;\text{hours}=\frac{\#injuries\;/\;\#games}{\#players\;on\;the\;ice\;at\;the\;same\;time\;*\#teams}*1000\;\text{games}$$

$$\text{IR}\;\text{per}\;1000\;\text{player}-\text{games}=\frac{\#injuries/\;\#games}{\#players\;on\;the\;ice\;and\;bench\;*\;\#teams}*1000\;\text{games}$$

To increase the data validity and to evaluate the data coverage, Game Injury Reports (GIR) were also completed on the same electrical database as the IRS forms by the health care staff of the team after each match. The GIR forms detailed the number of injuries sustained in a single game and were completed even if no injuries had occurred. The research team remained in contact with the teams to ensure data validity and to solve any possible problem situations that arose.

The injuries were classified by anatomic region, diagnosis, type, mechanism, penalization, and severity. The proportional distribution of injuries and the estimates of mean injury rates were calculated and studied using these classifications. The injuries and the mean injury rates were also classified and examined by player position, game period, and rink zone. When analyzing the proportions of specific injury details, injuries lacking this information were excluded from the single analysis.

## 3 Results

Injuries were collected for 1147 games by eight individual teams: 341 games by five teams in the 2017–18 season, 393 games by six teams in the 2018–19 season, and 413 games by seven teams in the 2019–20 season.

In total, 326 injury reports were documented in 1147 games during Liiga seasons 2017–2020. The average injury rate (IR) was 12.9 per 1000 player-games and 47.4 per 1000 player-game hours. The IR was the highest during the 2019–2020 season (131 injuries in 413 games; IR 14.4 per 1000 player-games) (Table 1).

**Table 1:** Number (n) and injury rates (IR) of injuries per 1000 player-games in total and annually.

Region	Total		2017–2018		2018–2019		2019–2020	
	n	IR	n	IR	n	IR	n	IR
**Head & face**	**118**	**4.7**	**33**	**4.4**	**43**	**5.0**	**42**	**4.6**
Head	51	2.0	11	1.5	24	2.8	16	1.8
Face	34	1.3	10	1.3	8	0.9	16	1.8
Teeth	29	1.1	10	1.3	10	1.2	9	1.0
Eye	2	0.1	1	0.1	1	0.1	0	0
Ear	2	0.1	1	0.1	0	0	1	0.1
**Upper extremity**	**89**	**3.5**	**22**	**2.9**	**31**	**3.6**	**36**	**4.0**
Shoulder	31	1.2	9	1.2	12	1.4	10	1.1
Hand	27	1.1	7	0.9	6	0.7	14	1.5
Wrist	16	0.6	1	0.1	10	1.2	5	0.6
Elbow	11	0.4	4	0.5	1	0.1	6	0.7
Forearm	4	0.2	1	0.1	2	0.2	1	0.1
**Lower extremity**	**84**	**3.3**	**25**	**3.3**	**25**	**2.9**	**34**	**3.7**
Knee	40	1.6	13	1.7	11	1.3	16	1.8
Foot	15	0.6	4	0.5	7	0.8	4	0.4
Thigh	8	0.3	3	0.4	1	0.1	4	0.4
Ankle	8	0.3	2	0.3	4	0.5	2	0.2
Hip/Groin	7	0.3	1	0.1	1	0.1	5	0.6
Leg	6	0.2	2	0.3	1	0.1	3	0.3
**Other**	**35**	**1.4**	**5**	**0.7**	**11**	**1.3**	**19**	**2.1**
Abdomen	8	0.3	3	0.4	2	0.2	3	0.3
Chest	7	0.3	0	0.0	3	0.3	4	0.4
Neck	6	0.2	0	0.0	3	0.3	3	0.3
Lower back	6	0.2	1	0.1	1	0.1	4	0.4
Pelvis	5	0.2	0	0.0	2	0.2	3	0.3
Genitals	2	0.1	1	0.1	0	0.0	1	0.1
Throat	1	0.0	0	0.0	0	0.0	1	0.1
**Total**	**326**	**12.9**	**85**	**11.3**	**110**	**12.7**	**131**	**14.4**

The head and face were the most commonly injured anatomical regions, accounting for 36.2% of all injuries (IR 4.7 per 1000 player-games). Most head and facial injuries were concussions (41.5%; IR 1.9), followed by lacerations (27.1%) and contusions (16.1%). Facial injuries comprised 20.6% of all injuries, and occurred mainly by a hit from a stick (43.3%) or a puck (26.9%). Dental injuries, which accounted for 8.9% of all injuries (IR 1.1 per 1000 player-games), were caused mainly by a hit from a stick (55.6%) or a puck (33.3%). Mouth guard use was reported in 63.2% of the dental injuries. Eye injuries were all lacerations and accounted for 3.0% of the facial injuries. A penalty was called in only half (48.1%) of the stick-caused facial injuries.

Of all injuries, 27.3% occurred to the upper extremity (IR 3.5 per 1000 player-games). The shoulder (IR 1.2) was involved in 34.8% of these injuries. In most shoulder injuries, the acromioclavicular (AC) joint (45.2%) or glenohumeral (GH) joint (35.5%) was affected. Shoulder injuries were associated with body checking in 76.7% of the cases, and 61.3% were related to board contact. Among the upper extremity injuries, hand and finger (30,3%) injuries were also common, as well as wrist injuries (18,0%). A hit by a puck was the most common etiology of the finger (50.0%) and hand injuries (80.0%). Body checking (56.3%) was the most common reason for wrist injury.

Lower extremity injuries accounted for 25.8% (IR 3.3 per 1000 player-games) of the injuries. Knee injuries accounted for 47.6% of the lower extremity injuries (IR 1.6 per 1000 player-games), followed by foot (15.5%), ankle (9.5%), and thigh (9.5%). Of the knee injuries, medial collateral ligament (MCL) injuries were the most typical diagnoses (51.6%). The anterior cruciate ligament (ACL) was rarely injured (6.5%). No specific diagnosis was documented in 22.5% of the knee injuries. Over half of the knee injuries were caused by body checking (54.1%).

**Fig. 1:**
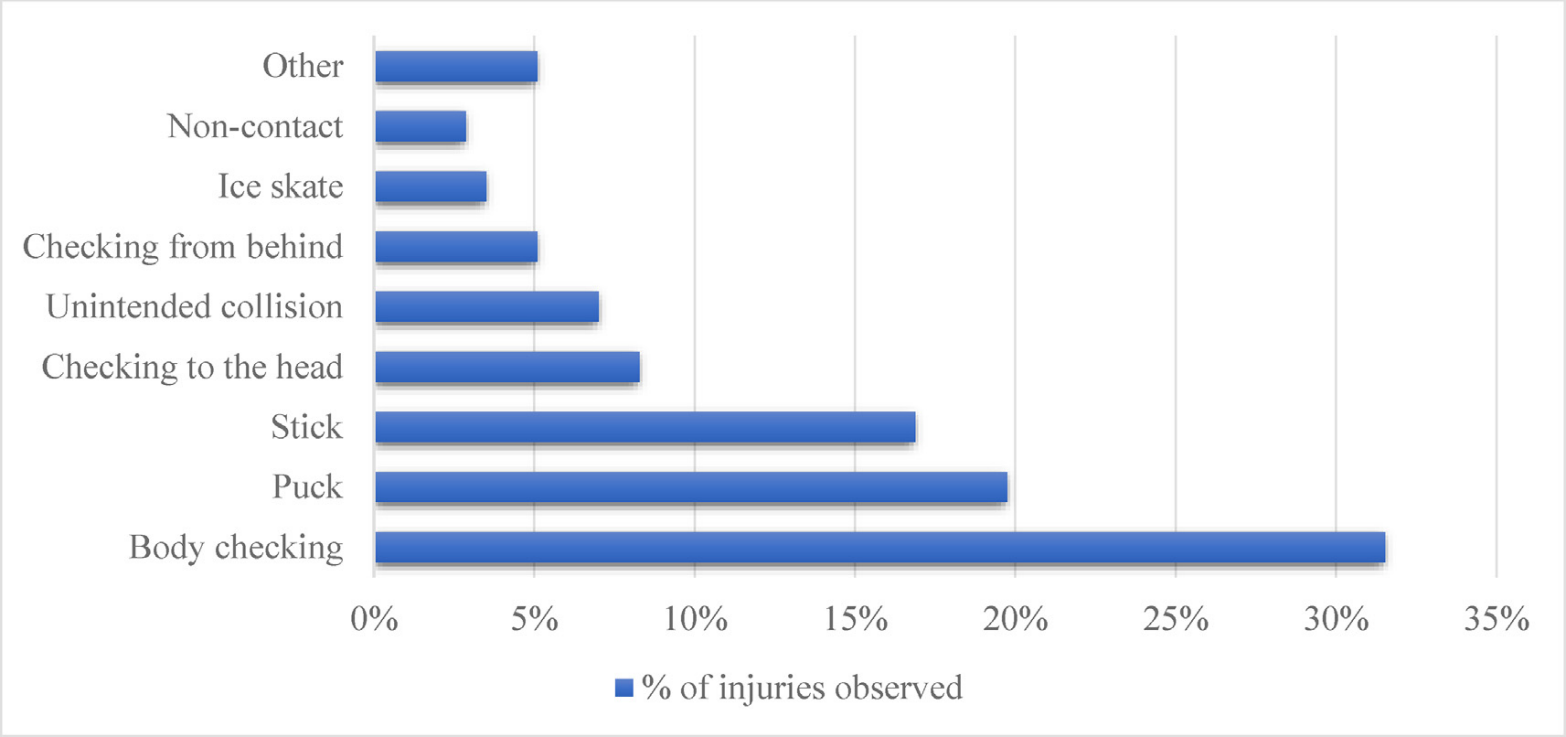
The causes of the injuries. In 3.8% of the cases, the cause of the injury was unknown.

**Fig. 2:**
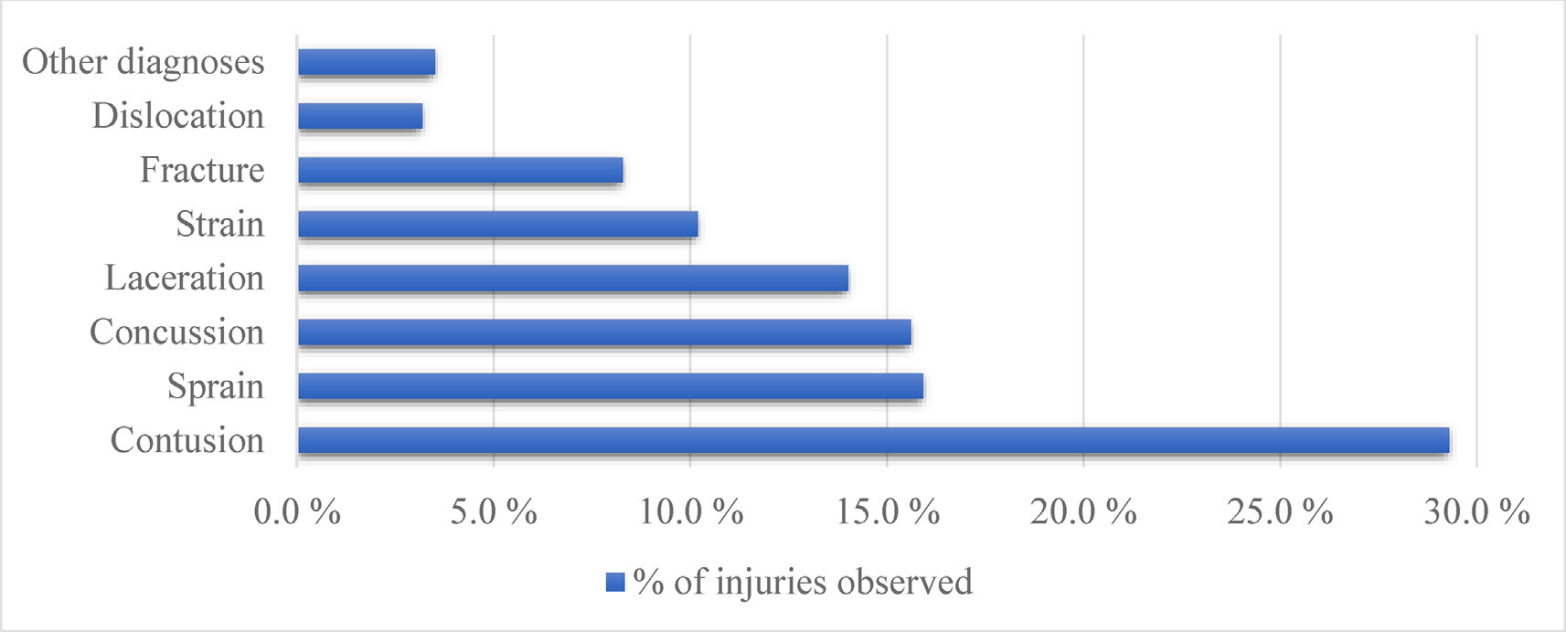
Injury types. In 3.8% of the injuries, the injury type was unknown.

Most injuries were caused by body checking (31.5 %, IR 3.9 per 1000 player-games), a hit by a puck (19.7 %, IR 2.5), or a hit by a stick (16,9 %, IR 2.1) (Fig. 1). A great majority (97.8%) of the injuries were acute in nature, and only 2.2% were recurrent from the current or previous seasons. The most common injury types were contusions (29.3%), sprains (15.9%), and concussions (15.6%) (Fig. 2). Among contusion injuries, a hit by a puck was the most common cause (46.2%), and the lower body (35.9%) was the most commonly injured body part.

**Fig. 3:**
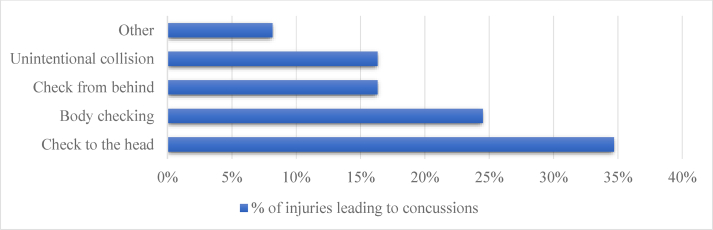
The mechanisms leading to concussions.

The rate of concussion was 1.9 per 1000 player-games (7.1 per 1000 player-game hours), accounting for 15.6% of the injuries. Over half of the concussions occurred due to a check to the head (34.7%) or a hit from behind (16.3%) (Fig. 3). Moreover, no penalty was enforced in 68.0% (76.5% and 50.0%, respectively) of these hits leading to concussions. The greatest risk for a concussion arose in the second period (50.0%), followed by the first period (33.3%), and then the third period (16.7%). The center players (24.5%) suffered the most concussions, followed by the defensemen (19.4% per defenseman), the wings (14.3% per wing), and the goalies (8.2%).

A minority of the injuries (29.7%, IR 3.7 per 1000 player-games) were related to board contact. However, 61.3% of shoulder injuries were related to board contact. In addition, 36.7% of the head injuries and 34.2% of the knee injuries were caused by contact with the boards.

**Fig. 4:**
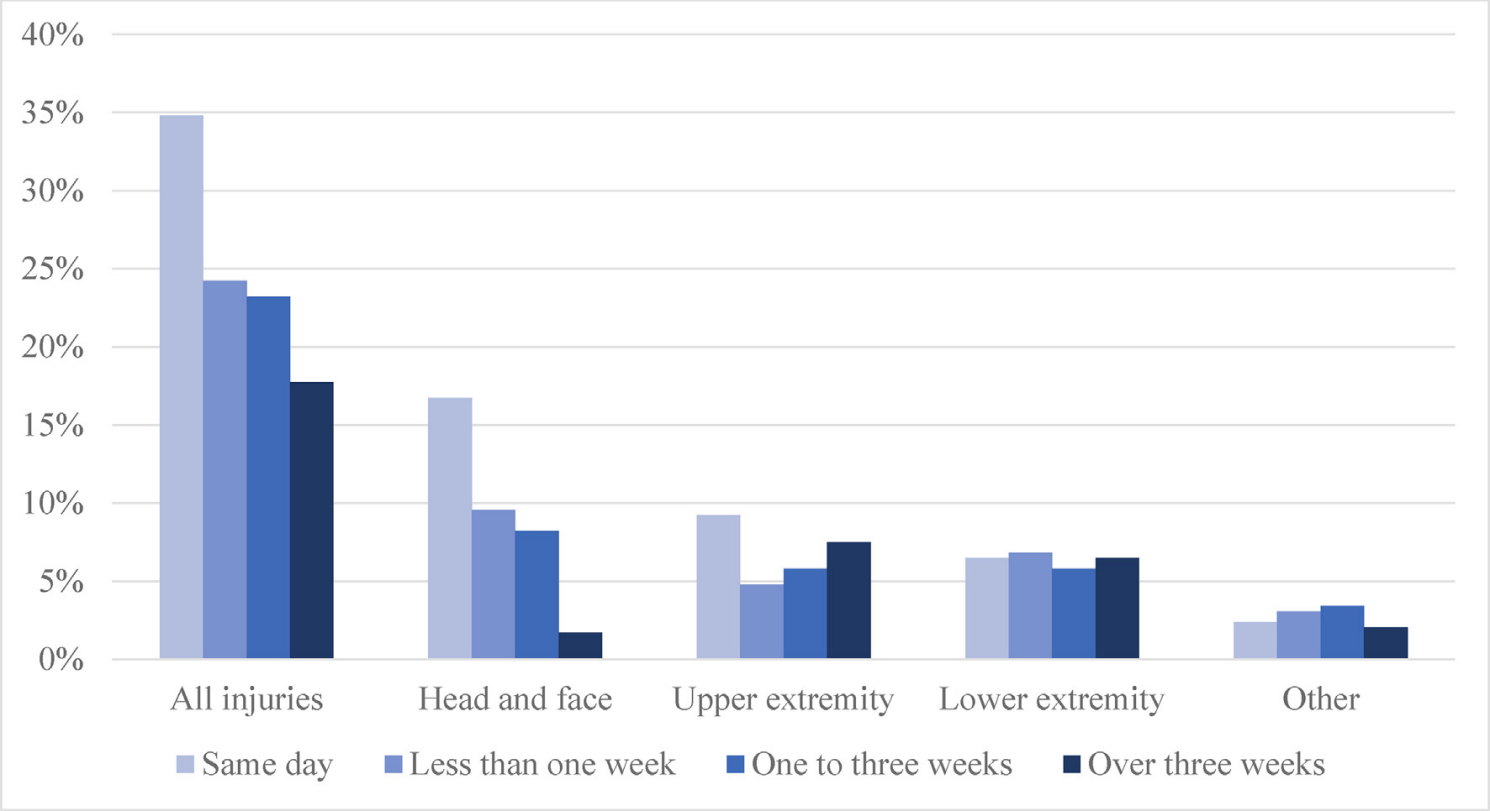
The time-loss of the injuries (% of injuries observed). In 10.1% of the cases, the severity of the injury was unknown.

**Fig. 5:**
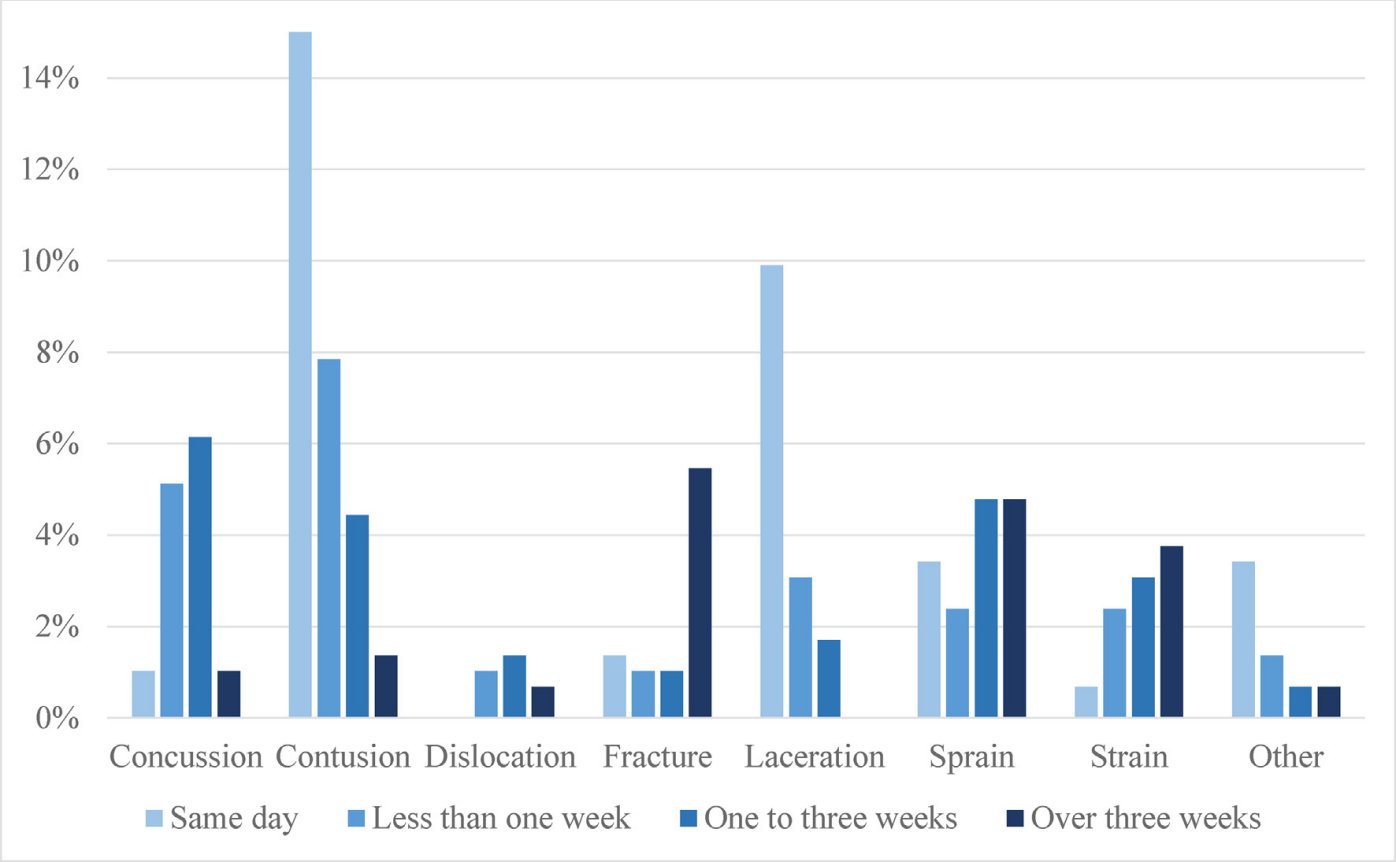
The time-loss caused by the different injury types (% of injuries observed). In 10.1% of the cases, the severity of the injury was unknown.

Recovery from injury took over three weeks in 17.7% of the injuries (IR 2.1 per 1000 player-games) (Fig. 4). These injuries were most commonly consequences of body checking (34.7%) or being hit by a puck (20.4%). Injuries related to a hit by a stick (6.1%), a check to the head (6.1%), or a check from behind (4.1%) seldom resulted in over three weeks of time loss. Knee (23.1%), hand and finger (21.2%), and shoulder (13.5%) injuries were the most frequently injured body parts resulting in over 3 weeks of time loss. An MCL injury was diagnosed in 75 % of the knee injuries requiring over three weeks recovery. In terms of injury types, fractures (30.8%), sprains (26.9%), and strains (21.2%) comprised most of the injuries requiring more than three weeks of recovery time (Fig. 5). Contusions (7.7%) and concussions (5.8%) represented a minority of these long-duration injuries.

The center was the player position with the highest injury incidence (22.4%; IR 2.9 per 1000 player-games), compared to the wing (19.2%; IR 2.5) and the defenseman (18.4%; IR 2.4). The goalie was rarely injured (2.5%, IR 0.3). The largest proportion of the injuries occurred in the second period (40.9%), followed by the first (30.1%) and third (28.4%) periods; however, this information was lacking in approximately a tenth of the cases. In total, 262 matches (22.8%) went to overtime [12] and 0.7% of the injuries occurred during the overtime period. The largest proportion of injuries occurred in the defensive zone (46.0%), followed by the offensive (40.0%) and the neutral (14.1%) zones.

## 4 Discussion

This research examined the ice hockey injury incidences and the details of the injuries during three consecutive seasons. Altogether, 326 injuries were documented in 1147 games, and the mean IR was 12.9 per 1000 player-games. The head and face were injured most frequently (36.2%; IR 4.7), while knee (IR 1.6) and shoulder (IR 1.2) injuries were also common. The typical mechanism of injury was body checking (31.5%; IR 3.9), in agreement with the existing literature [[6], [7], [8]]. The most common injury type was contusion (29.3%), while concussions (15.6%) were also sustained frequently. Remarkably, although half of the concussions occurred either due to checking to the head or from behind, penalties were called only in a minority (32.0%) of these cases. Hits by sticks caused most of the facial injuries, and again, penalties were not called in 51.9% of the stick-related facial injuries. Nearly one-fifth of all injuries (17.7%; IR 2.1) led to a time loss greater than three weeks; these were mostly fractures, sprains, and strains. The risk of injury was highest in the second period. Of all the players, the centers were injured most frequently (22.4%).

The main strengths of this study were the use of a web-based Injury Report System (IRS) form and the adoption of a competent injury definition that has been utilized previously in IIHF studies since 2006 [7,13,14]. In terms of data validity, the use of the Game Injury Report (GIR) forms improved the coverage of the data while providing an easy method for estimating the extent of under-reported injuries. The use of GIR forms also allowed the provision of reminders to the teams if injury reports were missing.

The main limitation of the research was that not all the Liiga teams participated in the reporting. Moreover, no simple tool was available to ensure that the teams were consistent with reporting and updating the details of injuries. However, the injury rates and types reported in this study are well in line with those of previous studies [6,7]. The effect of overtime and penalties on total athlete exposure was not taken into account; however, this had only a minor effect on the number of total exposure hours. In addition, the injury definition excluded injuries that did not cause time loss or require medical attention, regardless of their potential effect on the player's performance. According to the consensus statement by the IOC, the definition of injury could be broadened to include any complaint that negatively affects a player's performance [4].

Compared to previous studies, the IR was slightly lower than in men's IIHF tournaments (14.2 per 1000 player-games) and in the NHL (15.6 per 1000 player-games) [6,7]. However, in the NHL, the season is longer, the rinks are narrower, and fewer players comprise the squad. In the IIHF tournaments, the injury rate for shoulder (IR 1.7) and knee (IR 2.3) injuries was higher than in the present study (IR 1.2 and 1.6, respectively). This difference may reflect a higher intensity and pressure in the IIHF games, as well as the comprehensive use of flexible boards in Liiga. Compared to the other studies that considered injuries in the Finnish National League, conducted in the 1990s, the injury rate was 28.2% lower in the present study [10]. This lower injury rate may have arisen due to differences between the leagues, the eras, and the development of the equipment and the rules, as well as the slightly different study design and the definition of injury in the present study. Future research on ice hockey injuries is advised to use an IOC consensus on the definition of an injury and the injury rate [4].

Concussions persisted in frequency, accounting for 15.6% of all ice hockey injuries (IR 1.9 per 1000 player-games and 7.1 per 1000 player-game hours). Tuominen et al. [7] found an equivalent concussion rate for men's WC-tournaments (IR 1.9 per 1000 player-games), but the relative proportion of concussions was noticeably lower in their data (9.9% of all injuries). Flik et al. [15] reported a game-related proportion of concussions of nearly 23% of the injuries among American college ice hockey players. By contrast, Donaldson et al. [16] reported a concussion rate of only 0.52 per 1000 player-games in the NHL, whereas Benson et al. [17] presented concussion density as 1.8 concussions per 1000 player-hours in the NHL. Compared to previous studies in Finland in the 1990s, the relative proportion of concussions has risen 3.3-fold [10]. However, today, the diagnosis of concussions and brain damage may be faster and more accurate due, for example, to the development of the unified concussion protocol and the use of the Sport Concussion Assessment Tool [18,19]. Increased knowledge about concussions may also have led to a more cautious approach and lengthened player's time loss from the game after a concussion.

Illegal hits may be connected to higher measures of head impact severity compared to legal collisions [5]; consequently, they are frequent causes of injuries. In this research, a penalty was called in only 32.0% of the concussions caused by checks to the head and checks from behind. Thus, more accurate ways to prevent injuries should be pursued. With modern camera equipment, each injury situation can be rechecked from videos, and penalties could be called with the help of the off-ice officials. In our opinion, the health of players should be more important than the smooth flow of the game. Methods to reduce injuries could also include suspending players who accumulate numerous penalties during the season [20] and promoting fair play and sportsmanship more intensively [21].

## 5 Conclusion

In this three-year cohort study in Liiga, the injury rate (12.9 per 1000 player-games) was documented as slightly lower than the rates reported in previous studies that evaluated NHL and IIHF tournaments. The injury rate was also 28.2% lower than in previous studies conducted in Finland in the 1990s. The head and face were the body parts injured the most frequently, and body checking was the most common mechanism of injury. Concussions persisted as frequent injuries in elite male ice hockey. Over half of the concussions occurred due to an infraction. Recovery took over three weeks in 17.7% of the injuries, which of most were knee, hand or shoulder injuries. Infractions that led to injury were penalized in only a minority of the cases. Injury monitoring should be mandatory in all top-level leagues to improve the short-term and long-term health of the players.

**Key points**

• In Finland, the injury incidence among elite male ice hockey players has decreased compared to the 1990s. Injuries occur still frequently.
• Head injuries, especially concussions, persist to be frequent among elite male ice hockey players.
• Most common injury types requiring over three weeks recovery are fractures, strains, and sprains. Knee, hand (including fingers) and shoulder injuries comprise most of the long-term injuries.

## Ethics approval

As an anonymous register study, an ethics committee's review was not needed according to the criteria set by the Finnish National Board of Research Integrity TENK. All registrations and analyses were made anonymously.

## Funding

University of Jyväskylä has provided financial support to this research by paying the grammar check. However, the funding organization did not have any role in the collection of data, their analysis and interpretation, or in the right to approve or disapprove publication of the finished manuscript.

## Authorship

Jussi Hirvelä, Markku Tuominen and Jari Parkkari conceived and designed the study. All authors carried out data collection and contributed to interpretation of the data. Jussi Hirvelä performed literature search, data analyses and wrote the first draft of the paper. All authors revised it critically and contributed to the final manuscript. All authors have approved the submitted version of the manuscript and agreed to be accountable for all aspects of the work.

## Declaration of competing interest

None.
