# Supplementary material for: Acute injuries in male elite ice hockey players. A prospective cohort study
Source: JSAMS Plus. 2024 Jul 5;4:100068. doi: 10.1016/j.jsampl.2024.100068 (PMC13008420; doi:10.1016/j.jsampl.2024.100068)
Supplement: Multimedia component 1 [file mmc1.docx]

**SUPPLEMENTARY MATERIAL**


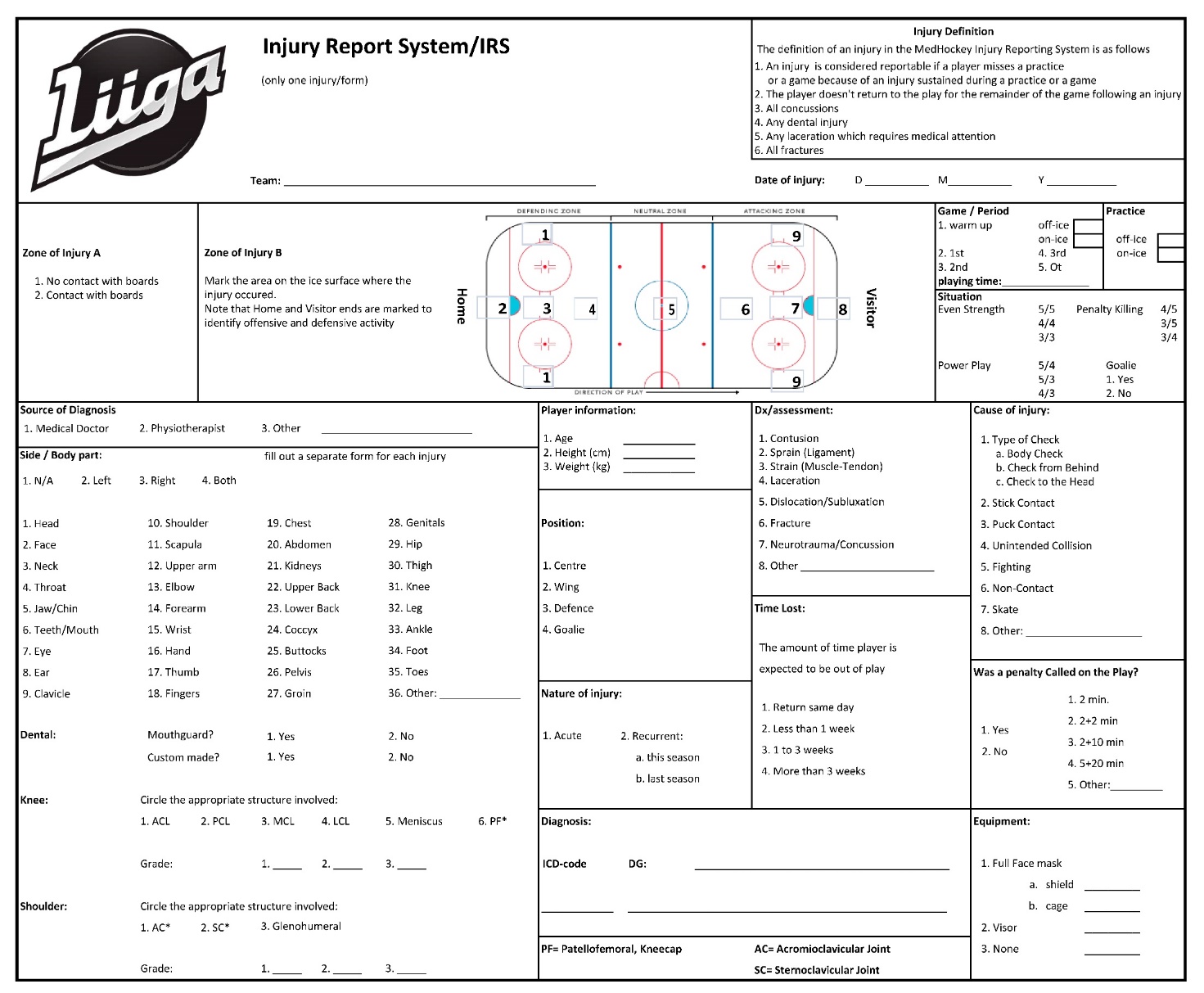


*The web-based Injury Report System (IRS) form was adapted as a paper version. Edited from the IRS form utilized in the IIHF studies, with permission from the authors*.
